# Empathy and moral judgment as psychological mechanisms of socially oriented consumption: evidence from an emerging economy

**DOI:** 10.3389/fpsyg.2026.1814181

**Published:** 2026-05-28

**Authors:** Alexander Sánchez-Rodríguez, Gelmar García-Vidal, Yandi Fernández-Ochoa, Rodobaldo Martínez-Vivar, Reyner Pérez-Campdesuñer

**Affiliations:** 1 Business Administration Research Group (GADEP), Universidad UTE, Quito, Ecuador; 2 Engineering Systems Research and Consulting Group (GICSI), Universidad UTE, Quito, Ecuador

**Keywords:** decision making, Ecuador, empathy, moral judgment, social cognition, socially oriented consumption decisions

## Abstract

**Introduction:**

Understanding how individuals translate concern for others into ethically oriented behavior remains a central challenge in moral and social psychology. This study examines whether empathy is associated with socially oriented consumption decisions and whether this relationship is statistically indirect through moral judgment as a core evaluative process. Drawing on social cognition and moral psychology, empathy is conceptualized as a higher-order psychological construct comprising cognitive and affective dimensions, whereas moral judgment refers to individuals’ evaluative assessments of fairness, responsibility, and harm in consumption contexts.

**Methods:**

The proposed model was tested using survey data from 1,000 adult urban consumers in Ecuador and analyzed through partial least squares structural equation modeling (PLS-SEM).

**Results:**

The results show that empathy is positively associated with moral judgment, and that moral judgment is positively associated with socially oriented consumption decisions. Empathy is also positively associated with socially oriented consumption decisions. The mediation analysis indicates a pattern consistent with partial complementary mediation, suggesting that moral judgment constitutes an important evaluative pathway linking empathy and socially oriented consumption decisions. The model shows moderate-to-substantial explanatory power and robust measurement properties.

**Discussion:**

These findings contribute to moral and social psychology by clarifying how cognitive and affective empathy are jointly associated with moral judgment and socially oriented consumption decisions in everyday consumption contexts. By providing large-scale empirical evidence from Ecuador, the study broadens the contextual basis of existing psychological research beyond predominantly Western settings and highlights the relevance of integrating affective and evaluative processes in the study of socially embedded consumer behavior.

## 1 Introduction

Human judgment and decision-making are fundamentally social processes. Even when individuals face decisions that appear personal or economically driven, such as everyday consumption choices, these decisions are often embedded in social contexts involving considerations of others’ welfare, moral norms, and collective responsibility. Contemporary research in behavioral and cognitive sciences increasingly recognizes that human decisions cannot be fully understood without accounting for the social-cognitive mechanisms through which individuals interpret social information, experience emotions toward others, and evaluate the moral consequences of their actions (Decety and Cowell, 2014; Zhang et al., 2025; Cameron et al., 2022).

From a social-cognitive perspective, decision-making extends beyond cost–benefit calculations or individual preferences. It involves integrating cognitive representations of others’ intentions, emotions, and social norms with affective responses and moral evaluations. In this context, empathy and moral judgment emerge as central processes linking social perception to behavioral choice. Empathy enables individuals to understand and emotionally resonate with others’ experiences, whereas moral judgment provides a cognitive framework for evaluating whether actions are right, fair, or socially acceptable (Kutaula et al., 2024; Hasan et al., 2023).

Consumption decisions offer a particularly relevant setting for examining these dynamics. Increasingly, consumers face choices with explicit social and moral implications, such as purchasing products associated with fair trade, local production, environmental sustainability, or support for vulnerable communities (Zhang et al., 2025). In such contexts, consumption becomes a site of moral evaluation, where individuals assess not only functional attributes but also the social consequences of their decisions (Alsaad, 2021; Yen and Yang, 2018).

Empathy plays a crucial role in this process by enabling consumers to cognitively and affectively connect with individuals or groups affected by their consumption decisions. Contemporary research conceptualizes empathy as a multidimensional construct comprising cognitive empathy—associated with perspective-taking—and affective empathy—related to emotional resonance (Cameron et al., 2022; Khalid and Dickert, 2022). Both dimensions facilitate the transformation of abstract social issues into personally meaningful evaluations (Karimzadeh and Boström, 2024).

Moral judgment constitutes a complementary evaluative process through which empathically salient concerns may become morally meaningful in decision-making. It refers to the evaluation of actions in light of moral standards such as fairness, responsibility, and harm avoidance, and emerges from the interaction between affective responses and higher-order reasoning (Decety and Cowell, 2014). In consumption contexts, moral judgment allows individuals to classify choices as acceptable or unacceptable, thereby guiding behavior (Alsaad, 2021; Hasan et al., 2023).

Recent literature emphasizes that empathy and moral judgment are not independent influences but mutually reinforcing processes. Empathy can intensify moral concern by making the consequences of actions more salient, while moral judgments can regulate empathic responses by determining who is perceived as deserving of care or blame (Cameron et al., 2022; Kutaula et al., 2024). This interaction highlights the importance of examining both constructs jointly when studying socially embedded decision-making.

A growing body of research links these mechanisms to prosocial and ethical consumption. Studies across cultural contexts show that empathy mediates the relationship between moral identity and ethical purchase intentions, and that empathic responses influence evaluations of socially responsible firms (Yang and Yen, 2018; Karimzadeh and Boström, 2024). However, despite these advances, existing research has tended to focus either on behavioral outcomes or on isolated psychological constructs, without explicitly modeling the integrated social-cognitive mechanisms that connect empathy, moral evaluation, and decision-making.

This limitation becomes more evident in non-Western and underrepresented contexts. Although prior studies have explored empathy and ethical judgment in countries such as Indonesia and Colombia (Arli and Anandya, 2018; Matallana et al., 2016; Chan et al., 2024), empirical evidence remains fragmented and insufficiently integrated into a unified explanatory framework. Moreover, the extent to which these mechanisms operate in other socio-cultural contexts remains poorly understood.

This gap is particularly relevant in Ecuador, where consumption decisions are embedded in social environments characterized by strong interpersonal ties, collective orientations, and a growing emphasis on social responsibility and sustainable consumption. Despite these contextual characteristics, there is a lack of large-scale empirical research examining how empathy and moral judgment jointly shape socially oriented consumption decisions in this setting.

Addressing these gaps, the present study examines how empathy is associated with socially oriented consumption decisions through moral judgment in the Ecuadorian context. Using survey data from urban consumers in Ecuador and partial least squares structural equation modeling (PLS-SEM), the study provides empirical evidence on the social-cognitive and moral-evaluative variables associated with ethically relevant consumption decisions.

By integrating empathy and moral judgment within a unified framework and situating the analysis in a non-Western context, this research contributes to a more comprehensive understanding of how social cognition shapes decision-making in socially and ethically relevant consumption contexts.

## 2 Literature review

### 2.1 Empathy as a social cognitive process

Empathy is a core construct within social cognition and refers to the capacity to understand and share others’ emotional and experiential states. Contemporary research conceptualizes empathy as a multidimensional process integrating cognitive and affective components (Cameron et al., 2022; Khalid and Dickert, 2022). Cognitive empathy involves perspective-taking and the ability to infer others’ intentions, emotions, and situational constraints, whereas affective empathy refers to emotional resonance and empathic concern for others’ well-being. These components operate jointly to facilitate social understanding and interpersonal evaluation (Hino, 2023).

Within moral psychology, empathy is widely recognized as a foundational mechanism underlying moral sensitivity and prosocial motivation. Empathic concern increases attention to the consequences of one’s actions for others, thereby shaping moral appraisals and ethical reasoning (Enginkaya and Sağlam, 2025). Individuals with higher empathic capacity tend to show heightened responsiveness to perceived harm and injustice, which influences how they evaluate morally relevant situations (Hino, 2023). Importantly, empathy is not merely an emotional reaction but a social-cognitive process that allows individuals to mentally represent others’ experiences and integrate this information into evaluative judgments (Rynarzewska et al., 2024).

Empirical research supports the importance of empathy in decision-making contexts that involve ethical considerations. Studies have shown that empathic individuals exhibit stronger moral concern and are more likely to engage in behaviors that promote others’ welfare, including charitable giving and ethical consumption (Batson, 2011; Yen and Yang, 2018). In consumer contexts, empathy has been found to motivate avoidance of products associated with harm and to increase support for socially responsible alternatives (Alsaad, 2021; Arli and Anandya, 2018; Huang et al., 2025). These findings suggest that empathy is theoretically relevant as a socially oriented disposition that shapes how individuals interpret the moral implications of consumption decisions (Rynarzewska et al., 2024).

Based on this theoretical and empirical foundation, empathy is expected to influence moral evaluation processes by increasing awareness of social consequences and activating concern for affected others. Thus, within the present framework, empathy is conceptualized as a socially oriented disposition that increases attention to others’ situations and to the possible consequences of one’s actions for their well-being. In this sense, empathy is theoretically relevant not because it directly determines behavior, but because it provides socially salient input that may later be interpreted through moral evaluation.

### 2.2 Moral judgment as an evaluative mechanism

In contrast to empathy, which concerns sensitivity to and understanding of others’ experiences, moral judgment refers to the evaluative appraisal through which actions and choices are interpreted in terms of fairness, responsibility, and harm. Within the proposed framework, moral judgment is therefore positioned not as a parallel construct, but as the evaluative domain through which empathically salient information may become normatively meaningful in consumption contexts.

Moral judgment is the cognitive process by which individuals evaluate actions, decisions, or outcomes in accordance with moral standards such as fairness, responsibility, and harm avoidance. Rather than being exclusively rational or emotional, moral judgment emerges from the integration of affective responses and higher-order cognitive reasoning (Decety and Cowell, 2014). In socially embedded decision contexts, moral judgment enables individuals to classify choices as morally acceptable or unacceptable and to justify their behavior accordingly (Tamaki, 2024).

In consumption settings, moral judgment plays a critical role in shaping decisions that carry ethical or social implications. Consumers frequently evaluate products and firms based on perceived ethicality, social responsibility, and alignment with moral values, and these evaluations significantly influence purchase intentions and behaviors (Tian et al., 2022; Tamaki, 2024). Moral judgment can thus be understood as an evaluative process associated with how empathically salient concerns become morally meaningful in consumption decisions.

Prior research indicates a robust association between empathy and moral judgment. Empathic concern enhances sensitivity to others’ suffering, which in turn increases moral condemnation of harmful actions and moral endorsement of prosocial alternatives (Cameron et al., 2022). Neurocognitive and behavioral studies demonstrate that individuals with higher empathy are more likely to consider intentions, consequences, and the impact on victims when making moral evaluations (Tian et al., 2022). These findings support the view that empathy provides an affective foundation for moral reasoning.

Moral judgment is also a well-established predictor of moral and prosocial behavior. Individuals who perceive a behavior as morally right or obligatory are more likely to act in accordance with that evaluation, even when doing so involves personal costs (Lu and Park, 2025; Enginkaya and Sağlam, 2025). In ethical consumption contexts, strong moral judgments increase commitment to socially responsible choices and reduce tolerance for unethical practices (Li et al., 2022). Accordingly, moral judgment is conceptualized in this study as an evaluative process statistically involved in the relationship between empathy and socially oriented consumption decisions (Huang et al., 2025).

### 2.3 Socially oriented consumption decisions

Socially oriented consumption decisions are those that explicitly incorporate concerns about social welfare, ethical responsibility, and the impact of consumption on others. These decisions extend beyond self-interested utility maximization and reflect an intention to contribute positively to society or to avoid causing harm through market behavior. Examples include purchasing fair-trade or locally produced goods, supporting socially responsible brands, or avoiding products associated with environmental or social harm (Li et al., 2022; Xin et al., 2025).

Consumption contexts differ in the extent to which they activate social-cognitive and moral processes. Not all consumption decisions involve the same level of moral salience or emotional engagement. For instance, utilitarian and routine purchases (e.g., basic goods such as food staples) tend to involve less moral reflection, whereas decisions about ethically differentiated products (e.g., fair-trade, sustainable, or socially responsible goods) are more likely to activate empathy and moral judgment. This distinction suggests that the influence of social-cognitive mechanisms may vary across consumption contexts, an aspect that remains underexplored in the literature. This distinction is particularly relevant when analyzing socially oriented consumption as a form of prosocial behavior in market contexts.

The literature conceptualizes socially oriented consumption as a form of prosocial behavior enacted within market contexts (Xin et al., 2025). Such behavior requires consumers to evaluate not only functional product attributes but also moral and social criteria, including fairness, responsibility, and social impact. As a result, socially oriented consumption is inherently linked to moral cognition and ethical evaluation.

Empirical studies across cultural contexts indicate that moral values and empathic concern are key drivers of socially responsible consumption (Chan et al., 2024). Consumers who experience stronger empathic responses toward affected individuals or communities are more likely to engage in ethical purchasing and boycott unethical firms (Yen and Yang, 2018). Research conducted in emerging economies further suggests that empathy and moral responsibility play a particularly salient role in shaping consumption decisions in contexts characterized by strong social ties and collective norms (Torelli et al., 2015; Tamaki, 2024; Lu and Park, 2025).

In this framework, socially oriented consumption decisions are positioned as the behavioral outcome of interest, reflecting the culmination of empathic engagement and moral evaluation.

### 2.4 Hypotheses development

Integrating the above theoretical arguments, this study proposes a social-cognitive framework in which empathy is associated with socially oriented consumption decisions both directly and indirectly through moral judgment. From this perspective, empathy is expected to be positively related to moral sensitivity, as individuals with greater empathic capacity may be more likely to attend to the social consequences of consumption and to interpret such decisions in morally relevant terms. Moral judgment, in turn, is expected to be positively associated with socially oriented consumption decisions, as individuals who evaluate these choices as fair, responsible, or morally appropriate may be more likely to align their consumption with such considerations.

Taken together, these arguments support a conceptually ordered framework in which empathy and moral judgment play distinct but related roles. Empathy is positioned as a socially oriented disposition associated with awareness of others and sensitivity to the interpersonal consequences of consumption, whereas moral judgment is positioned as the evaluative process through which such concerns are appraised in moral terms. This distinction provides the theoretical basis for examining whether empathy is associated with socially oriented consumption decisions both directly and indirectly through moral judgment. At the same time, the model does not assume that moral judgment is the only relevant intervening variable; rather, it focuses on one theoretically grounded pathway among others that may also be relevant, including moral identity, anticipated guilt, perceived consumer effectiveness, and social norms.

Consistent with prior research reporting positive relationships among empathy, moral evaluation, and prosocial behavior (Cameron et al., 2022; Decety and Cowell, 2014; Yen and Yang, 2018), the following hypotheses are proposed:

*H*1: Empathy is positively associated with moral judgment.

*H*2: Moral judgment is positively associated with socially oriented consumption decisions.

*H*3: Empathy is positively associated with socially oriented consumption decisions.

*H*4: Moral judgment statistically mediates the association between empathy and socially oriented consumption decisions.

These hypotheses are examined using partial least squares structural equation modeling (PLS-SEM), which is appropriate for analyzing complex relationships among latent social-cognitive constructs and for estimating indirect associations in predictive models.

### 2.5 Conceptualization of the measurement model

Consistent with the theoretical framework and the nature of the constructs examined, all latent variables in this study are conceptualized as reflective constructs. In reflective models, the latent construct is assumed to give rise to the observed indicators, such that changes in the underlying construct are reflected in variations across its measurement items (Hair et al., 2022).

Empathy is modeled as a second-order reflective construct composed of two first-order dimensions: cognitive empathy and affective empathy. Both dimensions represent manifestations of the broader empathic capacity and are therefore expected to covary. Cognitive empathy reflects individuals’ ability to take others’ perspectives and understand their experiences, whereas affective empathy captures emotional resonance and concern for others’ well-being. This specification is consistent with dominant conceptualizations of empathy in social cognition research (Cameron et al., 2022; Khalid and Dickert, 2022).

Moral judgment is conceptualized as a reflective construct capturing individuals’ evaluative assessments of moral appropriateness, fairness, and social responsibility in consumption contexts. Variations in moral judgment are expected to manifest consistently across items assessing perceived ethicality and moral obligation (Decety and Cowell, 2014).

Similarly, the construct of socially oriented consumption decisions is specified as a reflective construct representing the extent to which consumers’ choices are guided by social and ethical considerations. The observed indicators reflect an underlying disposition toward socially responsible consumption rather than its formation.

This reflective specification is theoretically justified by the assumption that the indicators are interchangeable manifestations of their respective latent constructs and are empirically expected to exhibit substantial intercorrelations. The reflective measurement model is therefore appropriate for subsequent assessment of reliability, convergent validity, and discriminant validity within the partial least squares structural equation modeling (PLS-SEM) framework (Hair et al., 2022).

**Figure 1:**
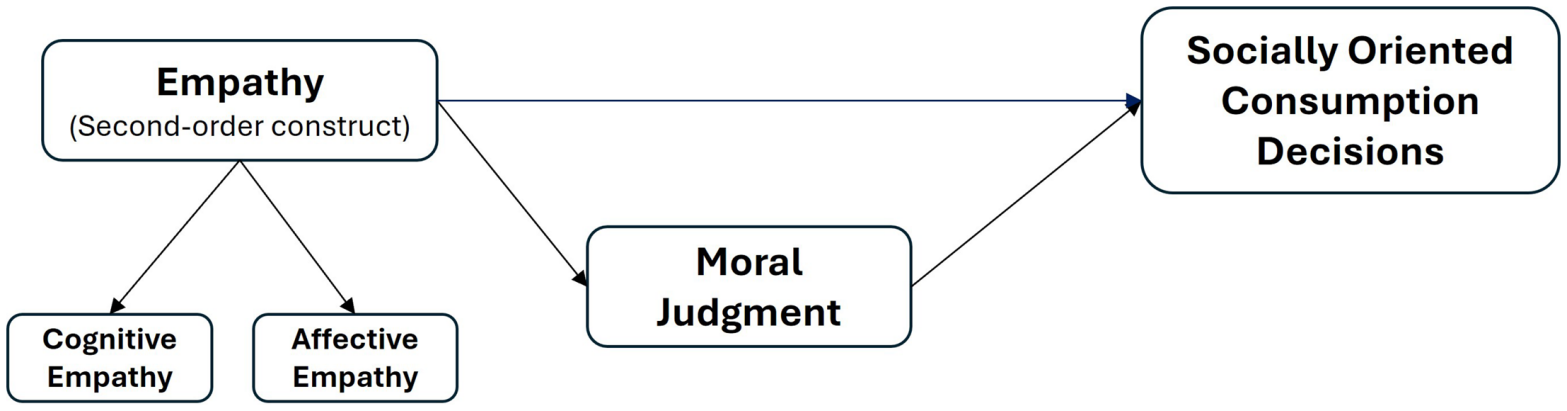
Conceptual model of empathy, moral judgment, and socially oriented consumption decisions. All constructs are specified as reflective latent variables and estimated using PLS-SEM.

Figure 1 illustrates the proposed social-cognitive framework, in which empathy—conceptualized as a second-order reflective construct comprising cognitive and affective dimensions—is associated with socially oriented consumption decisions both directly and indirectly through moral judgment.

## 3 Materials and methods

### 3.1 Research design

This study adopts a quantitative, cross-sectional research design to examine the relationships among empathy, moral judgment, and socially oriented consumption decisions in the Ecuadorian urban consumer context. A survey-based approach was selected because it is appropriate for examining theoretically grounded associations among latent constructs using structural equation modeling.

The analytical framework is based on partial least squares structural equation modeling (PLS-SEM). This technique is particularly suitable for research focused on prediction and theory development, latent construct modeling, indirect association analysis, and complex measurement structures, including higher-order constructs (Hair et al., 2022). Given the study’s objective of analyzing theoretically grounded relationships among latent constructs in socially embedded consumption contexts, rather than confirming an established covariance structure, PLS-SEM is methodologically appropriate.

### 3.2 Sample and data collection

The target population of this study consists of adult consumers aged 18 and older residing in Ecuador who regularly make consumption decisions involving social and ethical considerations. Given the socially embedded nature of empathy and moral judgment, the analysis focuses on urban consumers, for whom exposure to socially oriented consumption narratives, local production initiatives, and ethical consumption alternatives is more prevalent.

The analytical sample comprises 1,000 respondents residing in major urban areas of Ecuador. Data were collected using a structured online questionnaire administered to participants who voluntarily agreed to take part in the study. Prior to participation, respondents were informed about the academic purpose of the research, the anonymity of their responses, and the exclusive use of the data for scientific analysis.

The final sample exhibits a balanced gender distribution and is concentrated among the economically active age groups, consistent with the demographic profile of consumers who frequently face socially relevant consumption decisions. Most respondents report tertiary or postgraduate education levels and are employed either in the private sector or as self-employed workers, reflecting a population with regular exposure to market choices involving ethical and social considerations. Income and household size distributions correspond to typical urban consumption patterns, supporting the suitability of the sample for examining socially oriented consumption decisions.

The total sample size was set at *n* = 1,000 respondents to enhance statistical power, ensure estimator stability, and allow for robust testing of mediation effects within the proposed PLS-SEM framework. This sample size exceeds the recommended thresholds for structural equation modeling with latent constructs and is particularly appropriate for models that incorporate second-order constructs and indirect effects (Hair et al., 2022).

The sampling error was estimated using the standard finite population formula:


$$e=\sqrt{\frac{Z^{2}\mathrm{pq}(N-n)}{n(N-1)}}$$

Where e represents the sampling error, Z is the standard normal value corresponding to a 95% confidence level (1.96), p is the assumed proportion of success (0.5), q is the proportion of failure (0.5), N denotes the adult urban population size, and n is the sample size. Under these parameters, the estimated sampling error is approximately 3.1%, indicating high precision for multivariate behavioral analysis.

The sociodemographic characteristics of the analytical sample are summarized in Table 1. The final sample comprises 1,000 adult urban consumers and exhibits a balanced gender distribution, with 51.0% male and 49.0% female respondents. The age distribution is concentrated among economically active individuals, particularly those aged 25 to 60, who account for nearly 70% of the sample. This profile is consistent with population segments that regularly face consumption decisions involving social and ethical considerations.

**Table 1:** Sociodemographic characteristics of the analytical sample (*n* = 1,000).

Variable	Category	*n*	%
Gender	Male	510	51.0
	Female	490	49.0
Age group	18–25	165	16.5
	25–40	360	36.0
	40–60	335	33.5
	>60	140	14.0
Education level	Secondary or lower	85	8.5
	Technical / undergraduate	460	46.0
	Postgraduate	455	45.5
Employment status	Public employee	105	10.5
	Private employee	365	36.5
	Self-employed	255	25.5
	Student	155	15.5
	Unemployed / retired / other	120	12.0
Household size	1 person	115	11.5
	2–3 persons	355	35.5
	4–5 persons	385	38.5
	≥6 persons	145	14.5
Total sample		1,000	100.00

The sample consists of adult urban consumers residing in Ecuador. The distribution reflects economically active population groups that regularly engage in socially oriented consumption decisions.

In terms of educational attainment, the majority of respondents report tertiary or postgraduate education, reflecting a population with sufficient cognitive and informational resources to engage in moral evaluation and socially oriented decision-making. Employment status data indicate that most participants are employed in the private sector or self-employed, followed by students and public employees, a pattern consistent with typical urban labor-market structures.

Household size and income distributions further support the suitability of the sample for analyzing socially oriented consumption decisions. Most respondents live in households of two to five members, a structure commonly associated with shared consumption decisions and collective moral considerations. Overall, the sample composition shown in Table 1 reflects an economically active, socially exposed consumer group, providing an appropriate empirical basis for examining empathy-based moral judgment and socially oriented consumption decisions.

### 3.3 Measurement instrument

Data were collected using a structured questionnaire designed to measure empathy, moral judgment, and socially oriented consumption decisions. The instrument consists of 12 measurement items distributed across three first-order constructs and one second-order construct, as summarized in Table 2. All items were measured using a Likert-type scale, reflecting respondents’ level of agreement with statements related to social perception, moral evaluation, and consumption decisions.

**Table 2:** Measurement model: constructs, items, and sources.

Construct	Item code	Item wording	Source
Empathy (cognitive)	CE1	I understand how other people feel in consumption situations that affect them socially.	Cameron et al. (2022); Khalid and Dickert (2022)
	CE2	I can easily take the perspective of people who are affected by my consumption decisions.	
	CE3	I try to imagine how my consumption choices impact other people’s lives.	
Empathy (affective)	AE1	I feel concerned when my consumption decisions may negatively affect other people.	Cameron et al. (2022); Batson (2011)
	AE2	I feel emotional discomfort when I think my consumption choices could harm others.	
	AE3	I feel compassion for people who are negatively affected by irresponsible consumption practices.	
Moral judgment	MJ1	I believe that choosing socially responsible products is the morally right thing to do.	Decety and Cowell (2014); Alsaad (2021)
	MJ2	I feel morally obligated to consider the social consequences of my consumption decisions.	
	MJ3	I evaluate consumption choices based on whether they are fair and socially responsible.	
Socially oriented consumption decisions	SOC1	I prefer to buy products that have a positive impact on society, even if they cost more.	Yen and Yang (2018); Arli and Anandya (2018)
	SOC2	I avoid purchasing products from companies that harm society or vulnerable groups.	
	SOC3	Social and ethical considerations strongly influence my consumption decisions.	

All items were measured using a five-point Likert scale ranging from 1 (“strongly disagree”) to 5 (“strongly agree”). Empathy was modeled as a second-order reflective construct composed of cognitive and affective dimensions.

The construct of socially oriented consumption was operationalized using a multi-item scale capturing the extent to which individuals consider social and ethical implications in their consumption decisions, including concerns related to fairness, responsibility, and the welfare of others. Importantly, the measurement of socially oriented consumption captures a general behavioral tendency rather than behavior associated with a specific product category. Therefore, respondents were not asked about a particular type of good or service, but about their overall disposition to consider social and ethical aspects in their consumption decisions.

Empathy was operationalized as a second-order reflective construct composed of two first-order dimensions: cognitive empathy (three items) and affective empathy (three items). Cognitive empathy captures respondents’ ability to take others’ perspectives and to understand the social consequences of consumption decisions, while affective empathy reflects emotional concern and compassion toward individuals or groups that may be affected by irresponsible consumption practices.

Moral judgment was measured using three reflective items assessing respondents’ evaluative judgments regarding the moral appropriateness, fairness, and social responsibility of consumption choices. Socially oriented consumption decisions were also measured using three reflective items that capture the extent to which respondents’ purchasing behavior is guided by social and ethical considerations.

All items were adapted from previously validated measures used in prior research on empathy, moral judgment, and ethical or socially responsible consumption. The selection of items was guided by their conceptual alignment with the constructs examined in this study and by their prior empirical use in the literature. Specifically, empathy was operationalized through items capturing cognitive perspective-taking and affective concern; moral judgment through items reflecting fairness, responsibility, and ethical evaluation; and socially oriented consumption through items assessing the extent to which social and ethical considerations guide purchasing decisions. Because the instrument combines adapted items from different validated sources rather than reproducing a single original scale in its entirety, internal consistency was assessed directly in the present study through Cronbach’s alpha for first-order constructs, together with composite reliability and average variance extracted (AVE), in line with standard PLS-SEM practice. Minor wording adjustments were introduced to ensure contextual relevance and clarity while preserving the original conceptual meaning of each item. Responses were recorded using a five-point Likert scale ranging from 1 (“strongly disagree”) to 5 (“strongly agree”). Detailed results for first-order construct loadings, reliability statistics, and indicator significance are reported in Appendix A (see Tables A1, A2, and A4), while higher-order construct reliability and validity are presented in Table 3.

**Table 3:** Higher-order construct reliability and convergent validity.

Construct	Model specification	Composite reliability (CR)	AVE
Empathy	Second-order reflective (two-stage)	0.87	0.77

Given that empathy was modeled as a higher-order construct using the two-stage approach in PLS-SEM, reliability assessment focused on composite reliability and convergent validity (AVE), as Cronbach’s alpha is less appropriate for higher-order constructs.

Because the target population consisted of adult consumers in Ecuador, the questionnaire was administered in Spanish. As the original sources from which the items were adapted were published in English, a translation and back-translation procedure was conducted to ensure semantic equivalence and contextual appropriateness. First, the selected items were translated into Spanish while preserving their conceptual meaning. Next, an independent bilingual reviewer back-translated the Spanish version into English. The original and back-translated versions were then compared, and minor discrepancies were resolved through discussion within the research team. The final Spanish version was subsequently reviewed for clarity, face validity, and comprehensibility prior to full-scale data collection.

The questionnaire was structured to follow a logical cognitive sequence, beginning with empathy-related items, followed by moral judgment, and concluding with items related to socially oriented consumption decisions. This ordering was intentionally designed to reduce common method bias and to reflect the proposed causal flow of the conceptual model. All constructs were specified as reflective latent variables, and empathy was modeled as a higher-order construct in the PLS-SEM analysis.

Prior to full-scale data collection, the questionnaire was reviewed for clarity and face validity to ensure item comprehensibility and contextual appropriateness.

The measurement items were drawn from previously validated scales and reviewed for clarity and contextual relevance prior to data collection, ensuring the instrument’s appropriateness for the study context.

### 3.4 Data analysis procedure

Data analysis was conducted in two stages following standard PLS-SEM procedures (Hair et al., 2022). In the first stage, the measurement model was assessed to evaluate reliability and validity. Internal consistency reliability was examined using Cronbach’s alpha and composite reliability (CR). Convergent validity was assessed through the average variance extracted (AVE), with values exceeding the recommended threshold of 0.50 indicating adequate construct validity. Discriminant validity was evaluated using the heterotrait–monotrait ratio (HTMT). In addition, indicator reliability and significance were assessed through outer loadings and bootstrapping results. Detailed results for first-order constructs, including loadings, t-values, and *p*-values, are reported in Appendix A (see Tables A1 and A4).

In the second stage, the structural model was assessed to test the proposed hypotheses. Collinearity among predictor constructs was examined using variance inflation factors (VIF). Path coefficients were estimated and their significance evaluated using a bootstrapping procedure with 5,000 resamples. The explanatory power of the model was assessed using coefficients of determination (R^2^), while effect sizes (f^2^) were examined to evaluate the relative impact of each predictor. The significance of the structural relationships was assessed using bootstrapped standard errors, t-values, and confidence intervals.

Mediation effects were tested by examining the indirect effect of empathy on socially oriented consumption decisions through moral judgment. The significance of the indirect effect was assessed using bootstrapping, following current best practices in mediation analysis within PLS-SEM.

Empathy was modeled as a higher-order reflective construct using a two-stage approach. In the first stage, cognitive empathy and affective empathy were estimated as reflective lower-order constructs, and their latent variable scores were obtained. In the second stage, these scores were used as reflective indicators of the higher-order construct. This approach allows capturing the shared variance of the underlying dimensions while maintaining model parsimony and avoiding collinearity and identification issues associated with repeated-indicator specifications.

The use of PLS-SEM enables robust estimation of complex relationships among latent constructs and is particularly suitable for exploratory and predictive research in social cognition and decision-making.

### 3.5 Ethical considerations

The study adhered to established ethical standards for research involving human participants. Participation was voluntary, and informed consent was obtained prior to data collection. No personally identifiable information was collected, and all responses were treated confidentially and used exclusively for academic research.

## 4 Results

### 4.1 Measurement model assessment

**Figure 2:**
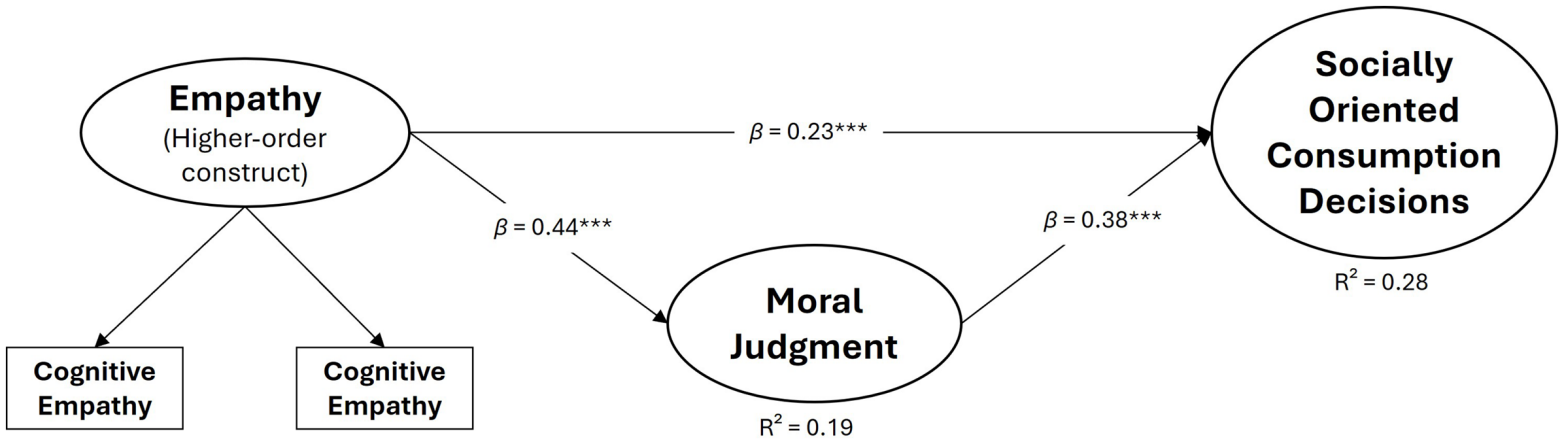
Structural model of empathy, moral judgment, and socially oriented consumption decisions. Empathy is modeled as a higher-order reflective construct comprising cognitive and affective empathy and is estimated using a two-stage approach. Standardized path coefficients (*β*) are reported for all structural relationships. Moral judgment partially mediates the relationship between empathy and socially oriented consumption decisions. Coefficients of determination (R^2^) indicate moderate explanatory power for moral judgment (R^2^ = 0.19) and socially oriented consumption decisions (R^2^ = 0.28). ***p < 0.001.

The measurement model was first assessed for indicator reliability, internal consistency, convergent validity, and discriminant validity. All constructs were specified as reflective, and empathy was modeled as a second-order reflective construct using a two-stage approach, consistent with the conceptual structure depicted in Figure 2.

For the first-order constructs—cognitive empathy, affective empathy, moral judgment, and socially oriented consumption decisions—all standardized outer loadings exceeded the recommended threshold of 0.70 and were statistically significant (*p* < 0.001) using the bootstrapping procedure, supporting indicator reliability. Internal consistency, reliability, and convergent validity were supported, as composite reliability values exceeded 0.70 and average variance extracted (AVE) values were above 0.50 for all first-order constructs, indicating that each construct explains more than half of the variance of its indicators (Fornell and Larcker, 1981; Hair et al., 2019). These criteria are consistent with standard PLS-SEM quality thresholds.

To ensure full transparency regarding the measurement quality of the lower-order constructs underlying the higher-order empathy construct, the complete set of first-order measurement results is reported in Appendix A. Specifically, Table A1 presents the standardized outer loadings, Table A2 reports internal consistency reliability and convergent validity statistics (Cronbach’s alpha, composite reliability, and AVE), and Table A4 provides the corresponding bootstrapped t-values and *p*-values, confirming the statistical significance of all indicators. Discriminant validity among first-order constructs is further supported by the HTMT ratios reported in Table A3, which remain below conservative cut-off criteria. Together, these results confirm that the first-order constructs are psychometrically sound and provide a robust empirical foundation for estimating the higher-order empathy construct.

In the second stage of the analysis, empathy was operationalized as a higher-order reflective construct using latent variable scores for cognitive and affective empathy. The reliability and convergent validity results for this higher-order construct are reported in Table 3, showing satisfactory composite reliability and AVE. In addition, Table 4 reports the higher-order construct loadings for affective empathy and cognitive empathy, confirming that both first-order dimensions contribute strongly to the higher-order empathy construct.

**Table 4:** Higher-order construct loadings.

Higher-order construct	Dimension	Loading
Empathy	Affective empathy	0.870
Empathy	Cognitive empathy	0.888

Discriminant validity among the main latent constructs was subsequently assessed using the heterotrait–monotrait ratio (HTMT). As shown in Table 5, all HTMT values were below the conservative threshold of 0.85, providing clear evidence that the constructs are empirically distinct. This criterion aligns with commonly used SmartPLS thresholds for assessing discriminant validity.

**Table 5:** Discriminant validity (HTMT criterion).

Construct pair	HTMT value	Validity
Empathy—moral judgment	0.52	Established
Empathy—socially oriented consumption decisions	0.47	Established
Moral judgment—socially oriented consumption decisions	0.49	Established

Taken together, the measurement model results indicate satisfactory reliability and validity, supporting its suitability for structural model evaluation.

### 4.2 Structural model results

Following the validation of the measurement model, the structural model was evaluated by examining collinearity, path coefficients, coefficients of determination (R^2^), and effect sizes (f^2^). Collinearity diagnostics indicated no multicollinearity concerns, as all variance inflation factor (VIF) values were below recommended thresholds.

The estimated structural relationships are reported in Table 6 and visually summarized in Figure 1. Bootstrapping results based on 5,000 resamples indicate that all hypothesized relationships are positive and statistically significant. Specifically, empathy is positively associated with moral judgment (*β* = 0.44, t > 10, *p* < 0.001), supporting H1. Moral judgment, in turn, is positively associated with socially oriented consumption decisions (*β* = 0.38, *t* > 10, *p* < 0.001), supporting H2. In addition, the direct association between empathy and socially oriented consumption decisions is also positive and statistically significant (*β* = 0.23, *t* > 10, *p* < 0.001), supporting H3.

**Table 6:** Structural model results.

Relationship	Standardized effect (β)	*p*-value	Hypothesis support
Empathy → Moral judgment	0.44	< 0.001	Supported
Moral judgment → Socially oriented consumption decisions	0.38	< 0.001	Supported
Empathy → Socially oriented consumption decisions	0.23	< 0.001	Supported

The model’s explanatory power is presented in Table 7. The model explains a moderate proportion of variance in moral judgment (R^2^ = 0.19) and a moderate-to-substantial proportion of variance in socially oriented consumption decisions (R^2^ = 0.28), indicating satisfactory predictive capability.

**Table 7:** Explanatory power of the structural model.

Endogenous construct	R^2^	Interpretation
Moral judgment	0.19	Moderate explanatory power
Socially oriented consumption decisions	0.28	Moderate–substantial explanatory power

To further assess the relative importance of the predictors, effect sizes (f^2^) were examined (Table 8). Empathy exhibits a medium-to-large effect on moral judgment, while moral judgment shows a medium effect on socially oriented consumption decisions. The direct association between empathy and socially oriented consumption decisions is comparatively small.

**Table 8:** Effect size assessment (f^2^).

Structural relationship	f^2^	Effect magnitude
Empathy → Moral judgment	0.24	Medium–large
Moral judgment → Socially oriented consumption decisions	0.17	Medium
Empathy → Socially oriented consumption decisions	0.06	Small

Overall, these results support all proposed hypotheses, as summarized in Table 9, and confirm the robustness of the structural relationships specified in the model.

**Table 9:** Mediation analysis results.

Effect type	Path	Standardized effect (β)	Significance
Direct effect	Empathy → Socially oriented consumption decisions	0.23	*p* < 0.001
Indirect effect	Empathy → Moral judgment → Socially oriented consumption decisions	0.17	*p* < 0.001
Total effect	Empathy → Socially oriented consumption decisions	0.40	*p* < 0.001

Bias-corrected 95% bootstrap confidence intervals excluded zero for all significant effects.

### 4.3 Mediation analysis

The indirect association of empathy with socially oriented consumption decisions through moral judgment (H4) was examined using a bootstrapping procedure with 5,000 resamples. The results are reported in Table 9.

The indirect association of empathy with socially oriented consumption decisions via moral judgment is positive and statistically significant (*β* = 0.17, *t* > 10, *p* < 0.001), and the corresponding bootstrap confidence interval excludes zero. These results indicate that the indirect association through moral judgment is statistically significant and consistent with the proposed mediation pattern.

At the same time, the direct association between empathy and socially oriented consumption decisions remains positive and statistically significant (*β* = 0.23, *p* < 0.001) when moral judgment is included in the model. This pattern is consistent with partial complementary mediation, insofar as both the indirect association through moral judgment and the direct association remain significant. Given the cross-sectional nature of the data, however, this result should be interpreted as evidence of a statistically consistent indirect association rather than as confirmation of causal or temporal mediation.

### 4.4 Summary of hypothesis testing

A consolidated summary of hypothesis testing results is presented in Table 10. All proposed hypotheses (H1–H4) are supported based on the estimated path coefficients and their statistical significance.

**Table 10:** Summary of hypothesis testing.

Hypothesis	Path	Result
H1	Empathy → Moral judgment	Supported
H2	Moral judgment → Socially oriented consumption decisions	Supported
H3	Empathy → Socially oriented consumption decisions	Supported
H4	Moral judgment mediates the relationship between empathy and socially oriented consumption decisions	Supported

The results confirm that empathy is positively associated with moral judgment, which in turn is positively associated with socially oriented consumption decisions. In addition, empathy is also positively associated with socially oriented consumption decisions, consistent with the proposed indirect association structure.

### 4.5 Robustness and model adequacy

The robustness of the results is supported by the large sample size (*n* = 1,000), the consistency of significance patterns across direct and indirect effects, and the absence of multicollinearity issues, as indicated by acceptable VIF values. In addition, all structural relationships are statistically significant and stable under bootstrapping procedures, reinforcing the reliability of the estimated parameters.

Model adequacy is further supported by the explanatory power of the endogenous constructs, as indicated by R^2^ values that suggest moderate to substantial predictive capability. Where applicable, predictive relevance (Q^2^) values greater than zero indicate adequate out-of-sample predictive relevance.

Furthermore, specifying empathy as a higher-order reflective construct using the two-stage approach ensures that both cognitive and affective dimensions are appropriately represented while maintaining model parsimony and avoiding estimation biases associated with more complex specifications. Additional robustness checks for the measurement and structural models are reported in Appendix A. Specifically, Table S5 presents the Fornell–Larcker criterion, which provides further evidence of discriminant validity, while Table S6 reports the Stone–Geisser Q² values, supporting the predictive relevance of the model for the endogenous constructs.

Overall, the results indicate that the model is empirically well-specified and provides a reliable basis for subsequent interpretation and discussion.

## 5 Discussion

The present study contributes to research on human judgment and decision making by jointly examining empathy, moral judgment, and socially oriented consumption decisions within a single analytical framework. The results indicate that empathy is positively associated with moral judgment and that both constructs are positively associated with socially oriented consumption decisions. Taken together, these findings are consistent with a process-oriented perspective in which affective and evaluative variables are jointly related to ethically relevant consumption decisions. Rather than claiming strong novelty, the study provides integrative evidence on these associations in a large sample from an underrepresented context.

### 5.1 Empathy as an antecedent of moral judgment

The results are consistent with the view that empathy is positively associated with moral judgment in socially oriented consumption contexts. The positive and statistically significant association between empathy and moral judgment (*β* = 0.44) suggests that individuals with greater capacity for perspective-taking and emotional resonance also tend to evaluate consumption choices more strongly through a moral lens. This interpretation is in line with contemporary models in moral psychology that conceptualize empathy as closely linked to sensitivity to harm, fairness, and responsibility by making the consequences of actions for others more salient (Decety and Cowell, 2014; Batson, 2011).

Importantly, operationalizing empathy as a higher-order construct comprising cognitive and affective dimensions provides a broader basis for interpreting this association. Cognitive empathy relates to individuals’ ability to mentally represent the experiences of others affected by consumption practices, while affective empathy reflects emotional concern and compassion. The positive association between this integrated empathic capacity and moral judgment suggests that moral evaluations in consumption contexts may be related both to understanding others’ situations and to affective concern for their well-being, rather than to abstract norm adherence alone.

This result is also consistent with prior empirical research that reports positive links among empathic concern, moral sensitivity, and ethical evaluation. At the same time, the present study adds further evidence from a large-scale sample in Ecuador, thereby contributing to a broader empirical base for examining empathy–morality linkages beyond predominantly Western, high-income settings (Culiberg et al., 2023; Hoffman, 2000).

### 5.2 Moral judgment as a central evaluative process

Moral judgment is positively associated with socially oriented consumption decisions and shows the strongest direct association in the model (*β* = 0.38). This finding suggests that moral evaluation is closely related to the extent to which social and ethical considerations are reflected in consumption choices. Consumers who perceive socially responsible consumption as morally right, fair, and obligatory may be more likely to report that such considerations are relevant in their purchasing decisions (Culiberg et al., 2023; Schwartz, 1977).

The magnitude of this association is consistent with theoretical perspectives that treat moral judgment as an important evaluative process in prosocial decision-making. From this perspective, when a behavior is cognitively classified as morally appropriate, individuals may be more inclined to align their conduct with that evaluation, even when doing so entails personal costs, such as higher prices or reduced convenience. This interpretation is compatible with norm activation theory, which emphasizes the relevance of moral obligation in prosocial behavior once individuals become aware of the consequences of their actions for others (Schwartz, 1977).

Within the broader human judgment literature, this result is consistent with the idea that moral cognition helps organize the incorporation of affective responses and contextual information into decisions. In this sense, moral judgment may be understood not simply as a reflection of empathic reactions but as an evaluative process shaped by how individuals interpret and organize those reactions in consumption contexts. This interpretation is also consistent with the indirect association results, which indicate that moral judgment is statistically involved in the relationship between empathy and socially oriented consumption decisions.

### 5.3 Direct and indirect associations of empathy with consumption decisions

The findings suggest a nuanced relationship between empathy and socially oriented consumption decisions. Empathy is positively associated with socially oriented consumption decisions through both a direct association (*β* = 0.23) and an indirect association through moral judgment (*β* = 0.17). The presence of both statistically significant paths is consistent with a pattern of partial complementary mediation, insofar as the association between empathy and socially oriented consumption decisions is not fully accounted for by moral judgment alone.

From a theoretical perspective, this pattern is consistent with the view that empathy may be related to socially oriented consumption decisions through multiple evaluative routes. On the one hand, empathy may be more directly linked to socially oriented consumption through emotional concern and identification with others affected by consumption practices. On the other hand, empathy may also be related to consumption decisions through moral judgment, insofar as empathic concern is associated with stronger moral evaluation of the social consequences of consumption.

This dual pattern is theoretically relevant because it aligns with broader debates in the literature over whether empathy is more closely tied to prosocial behavior through affective responsiveness or through its association with higher-order moral evaluation. Rather than favoring a single interpretation, the present results suggest that both types of association may coexist within the proposed framework.

Importantly, the persistence of a statistically significant direct association indicates that the relationship between empathy and socially oriented consumption decisions is not fully captured by moral judgment. At the same time, the significant indirect association suggests that moral judgment is meaningfully involved in this relationship. Given the cross-sectional nature of the data, however, these findings should be interpreted as statistically consistent with the proposed theoretical ordering rather than as evidence of distinct causal pathways.

Overall, these findings are consistent with integrative social-cognitive perspectives in which affective and evaluative processes are jointly associated with socially embedded decision-making. Rather than functioning as competing explanations, empathy and moral judgment appear in the present model as complementary constructs associated with socially oriented consumption decisions.

### 5.4 Implications for socially embedded decision making in the Ecuadorian context

The empirical context of this study—urban consumers in Ecuador—adds an important dimension to the interpretation of the findings. The model’s moderate explanatory power (R^2^ = 0.28 for socially oriented consumption decisions) suggests that empathy and moral judgment are relevant correlates of socially oriented consumption decisions in this context.

The Ecuadorian setting provides a relevant empirical context for examining how social-cognitive and moral-evaluative variables are related to consumption decisions. In socially embedded environments, consumption may be interpreted not only in individual terms but also in relation to broader concerns about responsibility, fairness, and effects on others. The positive association observed between moral judgment and socially oriented consumption decisions is consistent with this perspective and suggests that moral evaluation may be particularly relevant in contexts where consumption is closely linked to social meaning.

From a theoretical perspective, the present findings provide additional evidence from a non-Western setting to a literature predominantly developed in Western, high-income contexts. Rather than supporting strong comparative claims, the study broadens the empirical basis available for examining the relationships among empathy, moral judgment, and socially oriented consumption decisions. At the same time, the results highlight the importance of considering contextual factors when interpreting these associations and encourage future comparative research across different economic and cultural settings.

From a practical standpoint, the findings suggest that initiatives aimed at promoting socially oriented consumption may benefit from considering both empathic concern and moral evaluation. In particular, communication strategies that combine concern for affected others with ethical framing around fairness and responsibility may be more closely aligned with the psychological dispositions associated with socially oriented consumption in the present study. These implications should, however, be interpreted with caution, given the cross-sectional and self-reported nature of the data.

Finally, by providing large-scale empirical evidence from Ecuador, this study contributes to a more geographically diverse understanding of human judgment and decision making. The findings suggest that empathy and moral judgment are relevant constructs for studying socially oriented consumption in this context, while also underscoring the need to examine how these associations may vary across different social, cultural, and economic environments.

### 5.5 Theoretical contributions

This study makes incremental theoretical contributions to the literature on human judgment and decision making.

First, it contributes to social-cognitive research by jointly examining empathy and moral judgment within a unified analytical framework. Rather than treating these constructs as isolated predictors, the study analyzes their statistical relationships with socially oriented consumption decisions within a single model. In doing so, it highlights the relevance of considering both affective and evaluative dimensions when examining socially embedded decision-making.

Second, the study contributes to moral psychology by showing that moral judgment is significantly associated with both empathy and socially oriented consumption decisions, and that the indirect association between empathy and socially oriented consumption decisions, mediated by moral judgment, is statistically significant. These findings are consistent with process-oriented accounts of moral decision-making that emphasize the close relationship between affective dispositions, evaluative judgment, and behavior, while remaining cautious about causal interpretation.

Third, the research contributes to the literature on socially oriented consumption by adopting a process-oriented perspective rather than focusing exclusively on behavioral outcomes such as purchase intention. By modeling empathy as a higher-order construct and examining its direct and indirect associations with socially oriented consumption decisions, the study offers a more differentiated account of the psychological variables associated with this form of consumption.

Finally, by situating the analysis in Ecuador, the study broadens the empirical base of research on social cognition, moral judgment, and consumption beyond predominantly Western, high-income settings. Rather than establishing broad comparative claims, the findings provide context-specific evidence from an underrepresented setting and support the value of extending this line of inquiry to more diverse social, cultural, and economic environments.

### 5.6 Limitations and future research

While the present study provides relevant empirical evidence on the associations among empathy, moral judgment, and socially oriented consumption decisions, several limitations should be acknowledged, which also point to promising directions for future research.

A first limitation concerns the cross-sectional survey design adopted in this study, which does not permit strong causal inference. Although the proposed relationships are theoretically grounded and consistent with established social-cognitive perspectives, future research could employ longitudinal or experimental designs to more directly assess temporal ordering and causal direction. For instance, experimental manipulations of empathic salience or moral framing could help clarify how variation in these factors is associated with moral judgment and socially oriented consumption decisions over time.

A second limitation concerns the reliance on self-reported data for all constructs, which may introduce social desirability bias, particularly given the moral and ethical nature of the topics examined. Although the questionnaire was carefully designed to reduce evaluation apprehension and followed standard procedures for scale adaptation, future studies could complement self-reports with behavioral measures, such as choice tasks, field experiments, or revealed-preference data, in order to further assess the robustness of the observed associations.

From a contextual perspective, the empirical focus on urban consumers in Ecuador enhances the relevance of the findings for this setting, but limits the extent to which they can be generalized to rural populations or to other contexts with different cultural, institutional, or market characteristics. Social norms, moral narratives, and consumption infrastructures vary across societies, and these factors may condition the strength or configuration of the associations observed in this study. In addition, because the sample is urban, relatively highly educated, and based on online participation, it should not be interpreted as representative of the broader diversity of Ecuadorian consumers or of emerging economies more generally. Future research could therefore pursue cross-country comparative studies or multi-group analyses to examine how cultural values, levels of collectivism, or economic conditions may be associated with differences in these relationships.

Another relevant limitation stems from the lack of differentiation between types of goods or services and from the fact that respondents were not asked to report specific product or service categories in the questionnaire. As a result, the findings reflect a general disposition toward socially oriented consumption rather than behavior associated with particular consumption domains. Because different products and services may vary in moral salience and emotional engagement, future research should examine whether the associations observed here differ across categories such as utilitarian goods, experiential services, and ethically differentiated products.

Although empathy was modeled as a higher-order construct integrating cognitive and affective dimensions, the present study did not examine the potential differential associations of these dimensions at the structural level. Prior research suggests that cognitive empathy and affective empathy may relate differently to moral judgment and consumption decisions depending on context, target group, or moral domain. Future studies could therefore disaggregate these dimensions in the structural model to explore these possibilities more explicitly.

A related limitation concerns the model’s theoretical scope, which focuses on empathy and moral judgment but does not incorporate additional individual or contextual variables that may be relevant to socially oriented decision-making. Factors such as moral identity, perceived consumer effectiveness, social norms, trust in institutions, or economic constraints may interact with empathy and moral judgment in complex ways. In this sense, the present model should be understood as capturing one theoretically grounded pathway among several possible explanatory routes, rather than as an exhaustive account of socially oriented consumption decisions. Incorporating these variables could help refine the framework and provide a more comprehensive account of socially oriented consumption.

The study is also limited by the fact that it does not test competing theoretical models or boundary conditions. Accordingly, the findings should be interpreted as supporting one plausible framework rather than establishing the superiority of this specification over alternative explanations.

Finally, although the sample size and model specification provide a solid empirical basis for examining the proposed associations in this context, the focus on urban consumers limits the extent to which the findings can be generalized to the national population. The results should therefore be interpreted with caution when extrapolated to rural populations or to settings with different social, institutional, or market conditions.

Looking ahead, future research could extend the present framework beyond consumption contexts to other domains of socially embedded decision-making, such as organizational behavior, public policy support, or collective action. Examining whether similar patterns of association emerge across different decision environments would contribute to a broader understanding of social cognition and moral judgment in human decision-making.

In sum, while the current study provides useful evidence regarding the associations among empathy, moral judgment, and socially oriented consumption decisions, addressing these limitations through diverse methods, contexts, and theoretical extensions will further strengthen the understanding of how social-cognitive and moral-evaluative processes relate to judgment and behavior in ethically consequential settings.

## 6 Conclusion

This study contributes to research on human judgment and decision-making by examining how empathy and moral judgment are jointly associated with socially oriented consumption decisions in the Ecuadorian context. By modeling empathy as a higher-order construct and assessing its indirect association through moral judgment, the study offers a process-oriented account of how social concern relates to consumption decisions.

The findings indicate that empathy is positively associated with socially oriented consumption decisions both directly and indirectly through moral judgment. This pattern is consistent with partial complementary mediation, suggesting that moral judgment is an important evaluative process associated with the relationship between empathic disposition and socially oriented consumption, but does not fully account for the total association between these constructs. Given the cross-sectional design, these findings should be interpreted as theoretically consistent associations rather than causal effects.

By providing empirical evidence from Ecuador, the study contributes to a growing body of research that examines social-cognitive and moral-evaluative variables beyond predominantly Western settings. Rather than supporting broad comparative claims, the findings contribute context-specific evidence from an underrepresented setting and highlight the value of continuing this line of inquiry across diverse social, cultural, and economic environments.

Overall, the results underscore the importance of analyzing morally relevant decision-making as a domain in which emotional and cognitive components are closely related. The proposed framework offers a useful basis for future research on socially oriented behavior and highlights the relevance of moral evaluation in studying how psychological dispositions are associated with concrete consumption decisions.

## Data Availability

The raw data supporting the conclusions of this article will be made available by the authors, without undue reservation.
