# Supplementary material for: Empathy and moral judgment as psychological mechanisms of socially oriented consumption: evidence from an emerging economy
Source: Front Psychol. 2026 May 28;17:1814181. doi: 10.3389/fpsyg.2026.1814181 (PMC13254087; doi:10.3389/fpsyg.2026.1814181)
Supplement: Supplementary file 1 [file Supplementary_file_1.docx]

**Appendix A. Measurement Properties of First-Order Constructs**

Table A1. Indicator loadings for first-order constructs.

| Construct | Item | Item label (short description) | Loading |
| --- | --- | --- | --- |
| Cognitive empathy | CE1 | Emotional understanding | 0.824 |
|  | CE2 | Perspective-taking ability | 0.831 |
|  | CE3 | Impact imagination | 0.797 |
| Affective empathy | AE1 | Concern for others | 0.795 |
|  | AE2 | Emotional discomfort | 0.827 |
|  | AE3 | Compassion toward others | 0.805 |
| Moral judgment | MJ1 | Normative evaluation of responsible consumption | 0.833 |
|  | MJ2 | Moral obligation | 0.776 |
|  | MJ3 | Fairness-based evaluation | 0.777 |
| Socially oriented consumption decisions | SOC1 | Behavioral preference for responsible products | 0.826 |
|  | SOC2 | Avoidance of harmful products | 0.816 |
|  | SOC3 | Ethical influence on consumption | 0.806 |

Table A2. Construct reliability and convergent validity for first-order constructs.

| Construct | Cronbach’s alpha (α) | rho_A | Composite reliability (CR) | AVE |
| --- | --- | --- | --- | --- |
| Affective empathy | 0.737 | 0.738 | 0.851 | 0.655 |
| Cognitive empathy | 0.751 | 0.753 | 0.858 | 0.668 |
| Moral judgment | 0.710 | 0.717 | 0.838 | 0.633 |
| Socially oriented consumption decisions | 0.749 | 0.750 | 0.857 | 0.666 |

Table A3. HTMT ratios among first-order constructs.

| Construct pair | HTMT |
| --- | --- |
| Affective empathy – Cognitive empathy | 0.733 |
| Affective empathy – Moral judgment | 0.519 |
| Affective empathy – Socially oriented consumption decisions | 0.449 |
| Cognitive empathy – Moral judgment | 0.536 |
| Cognitive empathy – Socially oriented consumption decisions | 0.489 |
| Moral judgment – Socially oriented consumption decisions | 0.663 |

Table A4. Indicator significance (bootstrapping).

| Construct | Item | Item label (short description) | Loading | t-value | p-value |
| --- | --- | --- | --- | --- | --- |
| Cognitive empathy | CE1 | Emotional understanding | 0.824 | 18.42 | < 0.001 |
|  | CE2 | Perspective-taking ability | 0.831 | 19.10 | < 0.001 |
|  | CE3 | Impact imagination | 0.797 | 16.85 | < 0.001 |
| Affective empathy | AE1 | Concern for others | 0.795 | 16.72 | < 0.001 |
|  | AE2 | Emotional discomfort | 0.827 | 18.95 | < 0.001 |
|  | AE3 | Compassion toward others | 0.805 | 17.34 | < 0.001 |
| Moral judgment | MJ1 | Normative evaluation of responsible consumption | 0.833 | 19.88 | < 0.001 |
|  | MJ2 | Moral obligation | 0.776 | 15.62 | < 0.001 |
|  | MJ3 | Fairness-based evaluation | 0.777 | 15.80 | < 0.001 |
| Socially oriented consumption decisions | SOC1 | Behavioral preference for responsible products | 0.826 | 18.73 | < 0.001 |
|  | SOC2 | Avoidance of harmful products | 0.816 | 17.92 | < 0.001 |
|  | SOC3 | Ethical influence on consumption | 0.806 | 17.10 | < 0.001 |

Note. All indicator loadings are statistically significant at the 0.001 level based on bootstrapping results (5,000 resamples). Confidence intervals did not include zero.

Table A5. Discriminant validity (Fornell–Larcker criterion)

| Construct | Empathy | Moral judgment | Socially oriented consumption decisions |
| --- | --- | --- | --- |
| Empathy | **0.878** | - | - |
| Moral judgment | 0.52 | **0.796** | - |
| Socially oriented consumption decisions | 0.47 | 0.49 | **0.816** |

Note. Diagonal elements (bold) represent the square root of AVE. Off-diagonal elements represent inter-construct correlations.

Table A6. Predictive relevance (Q² values)

| Endogenous construct | Q² | Interpretation |
| --- | --- | --- |
| Moral judgment | 0.12 | Predictive relevance established |
| Socially oriented consumption decisions | 0.18 | Predictive relevance established |
